# Modality switching in children – is there an influence of modality compatibility?

**DOI:** 10.1007/s00426-025-02193-2

**Published:** 2025-10-14

**Authors:** Simone Schaeffner, Vera Wolfrum, Carina Lüke

**Affiliations:** Institute of Special Education, Chair of Special Education III – Special Education and Therapy in Language and Communication Disorders, Julius-Maximilians-University of Würzburg, Oswald-Külpe-Weg 84, 97074 Würzburg, Germany

**Keywords:** Input modalities, Output modalities, Semantic categorization, Gesture processing

## Abstract

Modality switching plays an important role in children’s language processing. During everyday life, especially during school hours, spoken language is often supplemented by visual information, resulting in frequent switching between auditory and visual information processing, as well as between the production of vocal and manual motor responses. Previous studies with adults have shown that modality switching is influenced by modality compatibility. Specifically, switching between incompatible mappings (i.e., auditory–manual and visual–vocal) leads to impaired performance, as reflected in higher mixing and switch costs, compared to switching between compatible mappings (i.e., auditory–vocal and visual–manual). So far, however, data are limited to adults, and underlying cognitive mechanisms are still under debate. The present study contributes to a better understanding by providing the first data from children. In two experiments, children switched between compatible and incompatible modality mappings while deciding whether the presented pictures and tones (Experiment 1; *N* = 32; *M*_age_ = 8.4 years), or gestures and spoken words (Experiment 2; *N* = 32; *M*_age_ = 8.4 years), represent an animal or not by pressing a yes- or no-button, or saying “yes” or “no.” Mixing costs were significantly higher for incompatible mappings than for compatible ones in both experiments. In contrast, switch costs were significantly influenced by modality compatibility in Experiment 2, but only marginally in Experiment 1. The results thus show that modality-specific effects on cognitive control processes already exist in childhood. Moreover, differences between the two experiments provide first evidence that these effects can vary depending on the type of input.

## Introduction

Switching between different modalities is of great relevance in children’s everyday lives – especially when they become pupils. A successful school day requires constant switching between auditory and visual processing when it comes to the perception and integration of spoken, written, or gestural information. In addition, it is often necessary to combine auditory or visual information processing with vocal or manual output, which can switch as well. For example, children listen to the teacher’s explanations and take notes (i.e., auditory–manual processing). Then, the teacher presents information visually on the board, and the children copy it down (i.e., visual–manual processing). While copying from the board, children may pause, briefly chat with each other, and then continue copying—switching from visual–manual to auditory–vocal processing and back. Although the ability to switch between different modality mappings as quickly and efficiently as possible is a decisive factor in meeting the demands of school, we know very little about how this ability develops, or about possible influencing factors, as our knowledge to date has been largely limited to modality switching in adults.

### Modality switching in adults

From studies with adults, it is well known that modality switching is influenced by modality compatibility (e.g., Friedgen et al., 2021; Schaeffner et al., 2016a, b; Stephan & Koch, 2010, 2016). Modality compatibility refers to the match between the input modality and the anticipated response effects of motor output. That is, auditory-vocal as well as visual-manual mappings are defined as compatible mappings because vocal output produces auditory effects, and manual output usually leads to visual effects. The two other combination options (i.e., auditory-manual and visual-vocal) are defined as incompatible due to the mismatch between the input modality and the anticipated response effects (see, e.g., Schacherer & Hazeltine, 2019, 2020).

A number of experiments demonstrated that the compatibility of modalities is particularly important when it comes to modality switching (Schaeffner et al., 2016a, b; Stephan & Koch, 2010, 2011). Switching between the two incompatible modality mappings (i.e., auditory-manual and visual-vocal) is slower, more error-prone, and, most importantly, leads to higher performance costs than switching between the two compatible mappings (i.e., auditory-vocal and visual-manual).

Performance costs in task or modality switching can refer to two different types of costs. On the one hand, there are switch costs in the form of longer reaction times (RTs) and higher error rates that are incurred when a task (or, in this case, a modality) has to be switched, compared to a task repetition (or modality repetition; for reviews, see Kiesel et al., 2010; Koch et al., 2018; Monsell, 2003). Switch costs are seen as a marker of cognitive control and are often understood as an expression of competition between the task sets (i.e., the mental representations of the tasks) to be switched, and the need to reconfigure the current task set (Monsell, 2003). On the other hand, there are mixing costs, i.e., performance costs that can even arise when a task or modality combination is repeated, as soon as the possibility exists that the task could change (i.e., in mixed-task or modality-switching conditions). Since performance is typically worse in mixed-task conditions than in single-task conditions (i.e., when only one task is performed), mixing costs are defined as the performance difference between repetition trials in mixed-task conditions (i.e., trials in which the task or modality mapping remains the same as in the previous trial) and trials in single-task conditions. Mixing costs are interpreted as a marker of increased working memory load, resulting from the need to maintain two task sets in parallel (e.g., Los, 1996), or from the uncertainty about which task has to be performed next (e.g., Rubin & Meiran, 2005; Poljac et al., 2009). Previous research has shown that both switch costs (e.g., Schaeffner et al., 2016a; Stephan & Koch, 2010, 2011), as well as mixing costs (e.g., Friedgen et al., 2021), can be influenced by modality compatibility.

An explanation for these differences in performance costs is the assumption of specific linkages between input modalities and compatible output modalities, due to the correspondence between the input modality and the sensory effects of compatible motor output. According to the ideomotor principle (James, 1890), it is assumed that actions are always controlled by the anticipation of the response effects that typically result from those actions. For example, vocal output in terms of saying “A” is guided by anticipating the auditory effect of hearing “A”. This means that, for switching between the two compatible modality mappings (i.e., auditory-vocal and visual-manual), the modality of the anticipated effect of motor output corresponds to the modality of sensory input in the current modality combination (e.g., in auditory-vocal processing, the anticipated auditory effect of vocal output corresponds to the input modality of the auditory-vocal task). In contrast, in the case of switching between incompatible modality mappings (i.e., auditory-manual and visual-vocal), the anticipated effect of motor output corresponds to the input modality of the *competing* modality mapping (e.g., in auditory-manual processing, the anticipated visual effect of manual output corresponds to the input modality of the competing visual-vocal task). This overlap is assumed to result in increased crosstalk, which leads to increased performance costs.

Crosstalk refers to potential conflicts that arise when performing one task produces overlap with another task. The exact mechanisms underlying crosstalk are still being debated. Recent research by Schacherer (2025) suggests that crosstalk in modality switching stems from interference between the representations engaged by central operations—specifically, from interference between the central codes, which include all task-relevant features (e.g., input modality, output modality, and anticipated response effects). During switching between the two incompatible modality mappings, these codes interfere because the anticipated response effect of one mapping interferes with the input modality of the competing mapping (see also Mueckstein et al., 2025, for first neural data), resulting in increased crosstalk. Higher crosstalk requires a higher level of cognitive control, which is reflected in higher switch costs when switching between incompatible mappings (Stephan & Koch, 2010, 2011, 2015, 2016). Similarly, the overlap between central codes increases task ambiguity and places a greater load on working memory during switching, resulting in higher mixing costs for incompatible mappings (e.g., Friedgen et al., 2021; Schacherer & Hazeltine, 2019; Schils et al., 2025).

Although the effects of modality compatibility are typically weaker for mixing costs than for switch costs, Friedgen et al. (2021) demonstrated that the effects on mixing costs can be amplified when verbal stimuli (i.e., the German words for “left” and “right” presented as written words on the screen or presented as spoken words via headphones) are processed instead of spatial stimuli (i.e., white diamonds presented to the left or right of the center of the screen or beep tones presented via headphones on either the left or right ear). They attribute this to a particularly strong connection between the auditory and vocal modalities during verbal processing. This leads to a strengthening of the auditory–vocal coupling, which, in turn, increases crosstalk and thus imposes a higher load on working memory when the two incompatible task sets must be maintained in an activated state while switching between incompatible modality mappings. As a result, modality-compatibility effects are stronger for verbal than for spatial processing. Further evidence for especially strong modality-compatibility effects in verbal processing was found in a study by Schaeffner and colleagues (2018b). It was shown that modality compatibility had a greater effect on switch costs during verbal processing (e.g., the processing of spoken or written words) than during nonverbal processing (e.g., the processing of nonverbal sounds or line drawings). This has been interpreted as reflecting stronger associations with potential response effects for verbal than for nonverbal input, which leads to increased crosstalk in incompatible modality mappings involving verbal input.

### Modality switching in children

Regarding modality switching in children, no study has yet investigated the effects of modality compatibility, and more general studies on modality switching in children (independent of the aspect of compatibility) are also rare. One of the few studies that did investigate modality switching in children was conducted by Ambrosi and colleagues (2011). They carried out a modality-switching experiment with seven-year-old children, who performed a property-verification task. In this task, the children were presented with terms such as “paintbrush,” “spoon,” or “ball”, along with associated properties like “has a handle”, “to eat yoghurt”, or “is thrown”. Children had to decide whether the property corresponded to the concept or not. Half of the properties were considered more motor-related, such as “is thrown”. The other half was considered more related to the visual modality, as it referred to an object’s global shape or to a part of it, such as “has a handle”. That is, in this study, children switched between modalities at the semantic level (i.e., switching between the semantic processing of motor-related or vision-related properties). However, the actual input and output modalities did not change, as both the concept names and the properties were presented bimodally—visually as written words on the screen, and auditorily as spoken words via loudspeakers. Thus, modality switching in this study involved more implicit switching at the semantic level, rather than explicit modality switching in terms of switching between listening (auditory modality), reading (visual modality), speaking (vocal modality), and typing or signing (manual modality). Nevertheless, even though children switched modalities only at the semantic level, they still showed faster responses for modality repetitions compared to modality switches, indicating modality switch costs.

More explicit modality switching, in terms of switching between listening to sounds and seeing pictures, was investigated in the study by Peng and colleagues (2018a). In their study, four- and six-year-old children, as well as adults, switched between an animal and a musical instrument detection task in a cross-modal task-switching paradigm. That is, animals and instruments were presented either auditorily (i.e., animal or musical sounds) or visually (i.e., pictures of musical instruments or animals), so that participants switched in an unpredictable sequence between auditory and visual input. The authors found significant modality switch costs that did not differ across age groups. At this point, however, it must be noted that—similar to the study by Ambrosi and colleagues (2011)—the output modality remained constant throughout the entire experiment (i.e., manual responses via a response key). This means that switch costs in this earlier study related exclusively to switching between input modalities and did not take into account the possibility of switching between output modalities.

To the best of our knowledge, no study has investigated children’s explicit modality switching that includes both input and output modalities, as well as input–output modality mappings. That is, our understanding of modality-specific effects on cognitive control is limited to adults’ modality switching, and it remains largely unknown whether modality compatibility plays a role in children’s modality switching. This is particularly noteworthy, as processing information from different input and output modalities is not only relevant for adults, but also for school-aged children, kindergarten children, and even in children who are just beginning to acquire language.

In early language development, for example, processing spoken language is often complemented by elements of visual communication, such as gestures (for an overview, see Rowe et al., 2022). Gestures are movements of the hands, arms, or other parts of the body that can occur either together with spoken language or instead of vocal speech production (McNeill, 1992). Previous studies have shown that combining spoken language and gestures is important in early language development as it allows children to express and understand things they could not yet manage with spoken words alone. For example, the use of gesture–word mappings enables children as young as 16 to 20 months to express two pieces of information in a single utterance (Capirci et al., 1996). This is approximately two months earlier than the ability to produce two-word utterances in spoken language (Iverson & Goldin-Meadow, 2005). Moreover, early communicative input from caregivers of young infants is characterized by a high degree of multimodality, combining gestures (visual input) and spoken language (auditory input; Lüke et al., 2017). This creates the need for infants to process different input modalities, and to respond using the limited productive skills they have developed so far, including both the manual output modality of gestures and the vocal modality of vocalizations.

Later, during the school years, processing language via different input and output modalities becomes increasingly important, especially during written language acquisition. It is well known that the ability to combine visual information with auditory or phonological information, in terms of cross-modal associations, is an important predictor of reading abilities (e.g., Hulme et al., 2007; Jones et al., 2013), and specific reading disabilities (“dyslexia”; for a review, see Roitsch & Watson, 2019) are discussed with regard to modality-specific aspects. Children must learn to combine spoken and written language, switching between listening to auditory input (i.e., spoken language), translating it into manual output (i.e., writing), and translating visual input (i.e., written text) into verbal output (i.e., reading aloud). Therefore, the question of whether children’s modality switching is influenced by modality compatibility is of great importance when trying to understand mechanisms of cognitive control, as well as the cognitive processes underlying children’s language processing.

### Research question and hypotheses

In order to clarify whether children’s modality switching is influenced by modality compatibility, we conducted two modality-switching experiments. In both experiments, children had to switch between compatible and incompatible input-output modality mappings while categorizing concepts as “animal” versus “no animal.” Children in both experiments gave yes- or no-responses (i.e., “yes” for “animal” and “no” for “no animal”) either by saying the German words “JA (yes)” or “NEIN (no)” (i.e., vocal output) or by pressing a yes- or no-key (i.e., manual output). The decisive difference between the two experiments lay in the presentation of the concepts. In Experiment 1, the concepts were presented as typical sounds, such as the meowing of a cat or the ticking of a clock (i.e., auditory input), or as line drawings (i.e., visual input). In contrast, in Experiment 2, the rather nonverbal concept presentation from Experiment 1 was replaced by a more verbal form of presentation. Specifically, concepts were presented as spoken words (i.e., auditory input presented via loudspeaker) or as gestures (i.e., visual input presented as short videos on the screen). The semantic categorization and required responses were identical to those in Experiment 1. If children’s modality switching is already influenced by modality compatibility similarly to adults (e.g., Schaeffner et al., 2016a), we expect higher performance costs for switching between incompatible mappings compared to switching between compatible mappings in both experiments. Furthermore, if the effects of modality compatibility are especially pronounced for the processing of verbal input (e.g., Friedgen et al., 2021; Schaeffner et al., 2018b), we expect stronger effects of modality compatibility in Experiment 2 compared to Experiment 1.

## Experiment 1

Experiment 1 represents the first modality-switching experiment in which children switched between different input-output modality mappings. Assuming that switching modality mappings leads to a higher working-memory load than constant processing of just one single modality mapping, we expect to find mixing costs. Similarly, we expect to find switch costs whenever children have to switch from one modality mapping to another because modality switches require a higher degree of cognitive control than modality repetitions. Moreover, if children are confronted with increased crosstalk when switching between incompatible modality mappings, we expect longer RTs, higher error rates, and—above all—higher mixing costs as well as higher modality switch costs for incompatible compared to compatible mappings, similar to findings from previous modality-switching experiments with adults. (e.g., Friedgen et al., 2021; Schaeffner et al., 2016a, 2018a; Stephan & Koch, 2010, 2011).

### Method

#### Participants

A total of 32 children^1^ (*M*_age_ = 8.4 years; *SD* = 1.2 years; range = 6.1–10.7 years; gender = 14 female) participated in Experiment 1. All children were German speakers, and no child had experience with sign language. Thirty of them were right-handed. Parents reported no neurological, psychiatric, or developmental disorders, as well as normal hearing acuity, and normal or corrected-to-normal visual acuity. Parental consent was obtained for all participants. The ethics board of the German Educational Research Association (GERA) approved the study. Children were recruited via flyers in primary schools, sports, or afternoon clubs, and the families received 30 Euros each as compensation for their participation in this study.

#### Task and procedure

Each child was tested in three different sessions with a maximum of one session per day. The time interval between sessions ranged from 1 to 23 days (*M* = 7 days). All three sessions took place at the Laboratory for Communication and Language at the University of Würzburg. The first session (Pretest) lasted approximately 60 min and was used to establish contact between the child and the experimenter, as well as to evaluate language abilities using standardized tests. We conducted two subtests of the P-ITPA (Esser & Wyschkon, 2010) to assess productive lexical and grammar skills, the German version of the TROG-D (Fox-Boyer, 2020) to assess receptive grammar skills, and the Mottier Test (Wild & Fleck, 2013) to assess phonological working memory. In this way, we aimed to ensure that only children with typical language development took part in the study.

**Fig. 1:**
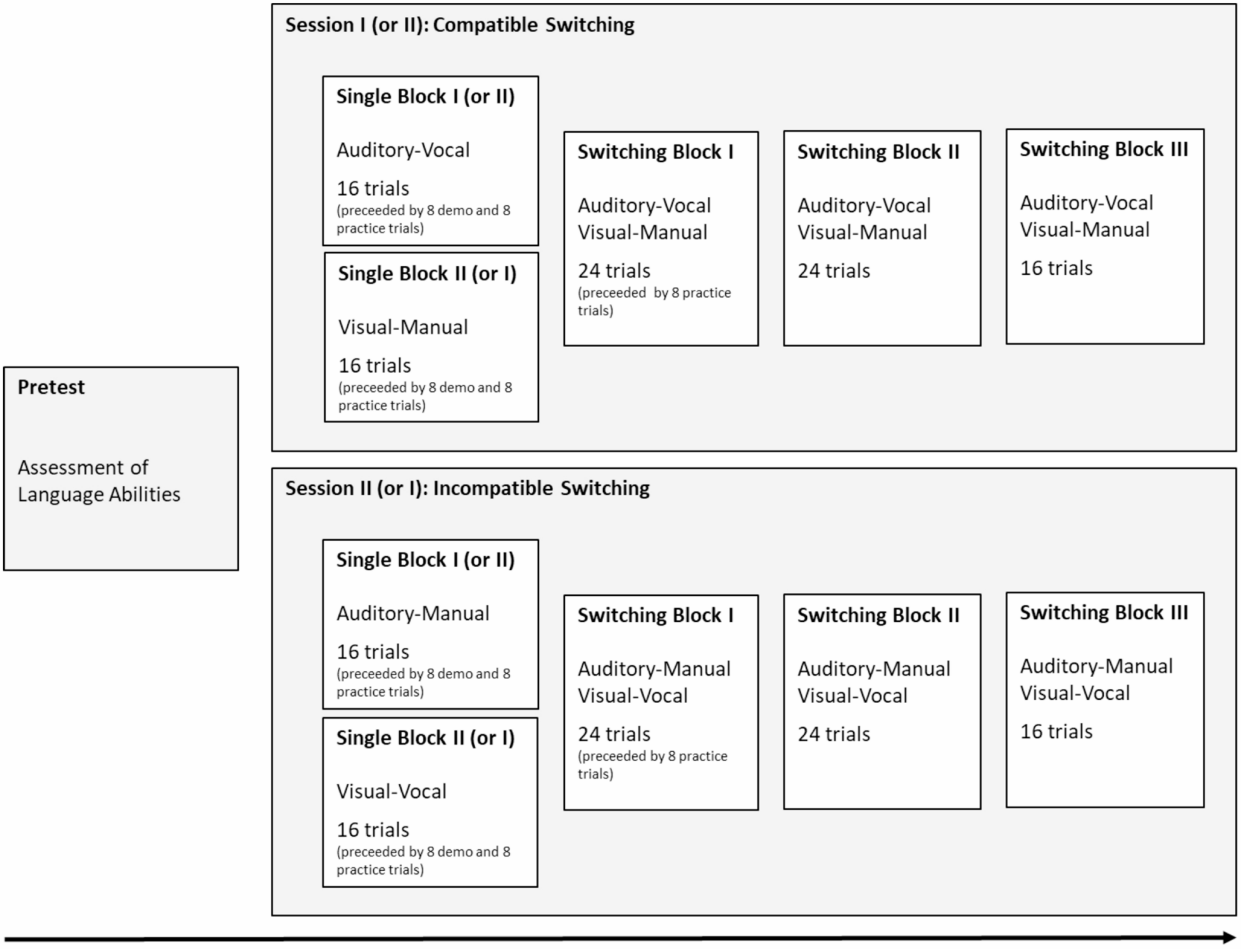
Schematic representation of the different sessions and blocks. The order of the compatible and incompatible switching session as well as the order of the two single blocks within the switching sessions was counterbalances across participants. Please note that the practice trials in single blocks could be repeated if the child was still very unsure about the task or if there were more than 50% incorrect responses

Parents were asked to complete a questionnaire about the child’s age, handedness, possible diagnoses, and therapies while their child was attending the Pretest. The other two sessions (Session I and II; see Fig. 1) lasted about 25–30 min each and consisted of game-based modality-switching tasks at a PC, in order to assess children’s modality switching. In one of the two modality-switching sessions (Session I or II), children switched between the two compatible modality mappings (i.e., auditory input combined with vocal output, and visual input combined with manual output); in the other session, children switched between the two incompatible mappings (i.e., auditory input combined with manual output, and visual input combined with vocal output). The decision to hold the sessions over two days was made because pilot tests showed that many children were unable to complete both the compatible and incompatible switching sessions in one day. The order of the compatible and incompatible switching sessions was counterbalanced across participants. Figure 1 shows a schematic representation of the three experimental sessions.

The modality-switching tasks of Session I and II were programmed and presented using E-Prime 3.0. In both sessions, children sat in front of a 14-inch laptop (viewing distance approximately 60 cm), performing a semantic categorization task. They were instructed to decide whether the presented concepts represented an animal or not by saying the German words “JA (yes)” or “NEIN (no)”, or by pressing a yes- or no-key. The concepts to be categorized were “bird”, “cat”, “cow”, “elephant”, as well as “baby”, “car”, “clock”, and “telephone”. The selection of concepts was controlled for concreteness, the number of syllables, and the word frequency of the corresponding German words.

The eight concepts were presented as auditory or visual stimuli, depending on the current experimental block (for an overview of the different blocks, see Fig. 1). Auditory stimuli consisted of typical sounds associated with the concepts, such as the meowing of a cat or the ticking of a clock. The sounds were presented with controlled durations (between 900 and 2200 ms) via two external speakers, which were placed symmetrically to the right and left of the PC, with a distance of 1.20 m between them, and had an average volume of 55 dB.

Visual stimuli were line drawings (size: 512 × 512 pixels), displayed on a white background at the center of the monitor. The line drawings were presented until a response was made or for a maximum duration of 4000 ms. To ensure that the sounds and line drawings could be clearly assigned to the intended concepts, 65 independent adult raters evaluated them in advance. The auditory or visual presentation of the stimuli was combined with vocal or manual responses, depending on the current block (see Fig. 1). Vocal responses (i.e., the words “JA [yes]” and “NEIN [no)]”) were given via a microphone, which was connected to the E-Prime response box “Chronos”. Manual responses were made by pressing the left or right response button of the response box with the index finger of the left or right hand. The two buttons were previously marked as yes- and no-buttons using a green tick and a red cross, with the left–right allocation counterbalanced across participants. Children were instructed to always respond as quickly and as accurately as possible. The accuracy of vocal responses was coded online by the experimenter, and the accuracy of manual responses was recorded by the computer. The PC also recorded RTs for both vocal and manual responses.

**Fig. 2:**
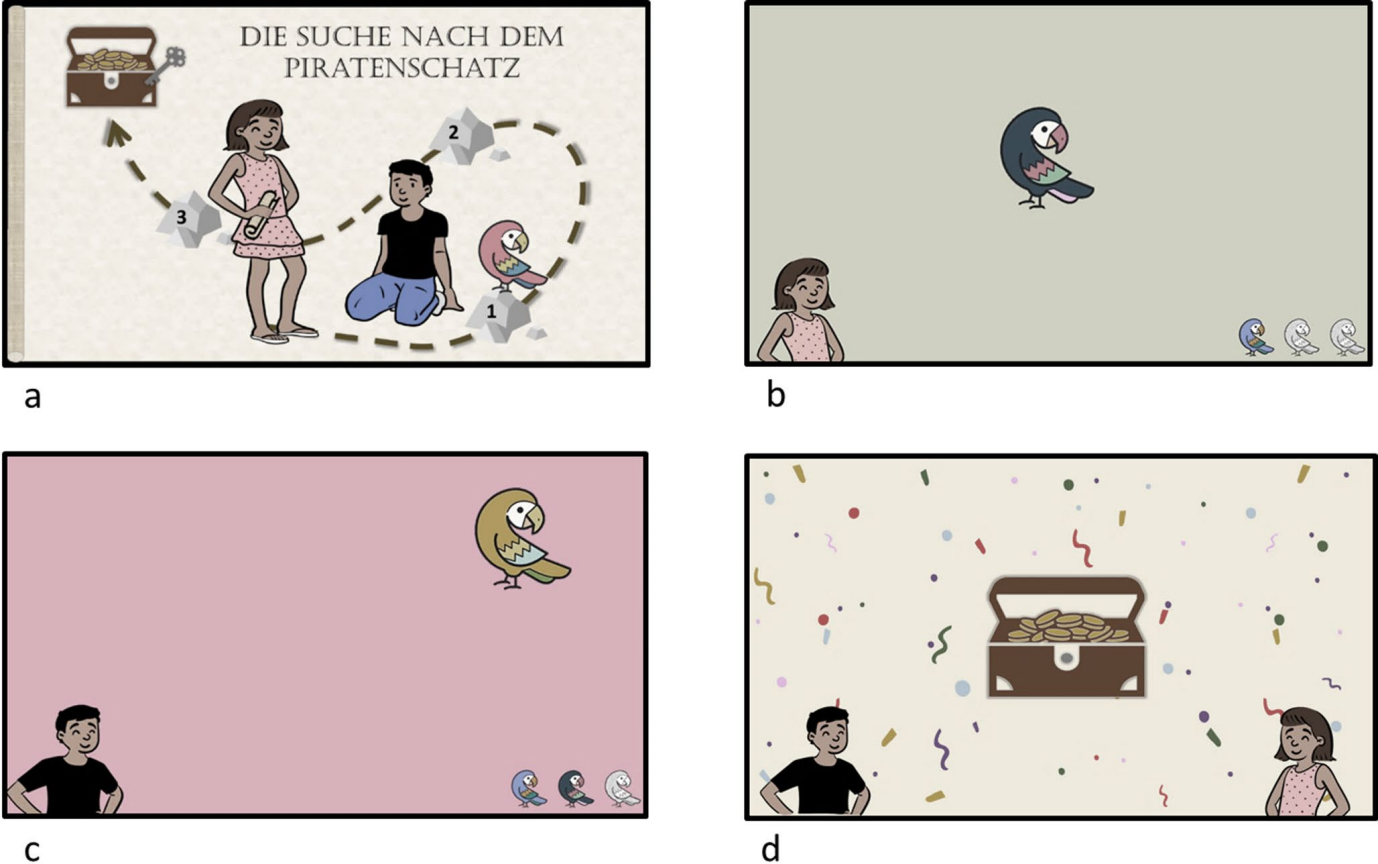
Selected pictures of the cover story. The introduction of the story “The Search for Pirate Treasure” and the two protagonists Anna and Paul (**a**). Feedback from the protagonist Anna with regard to the progress of the blocks already completed (**b**). Feedback from the protagonist Paul with regard to the progress of the blocks already completed (**c**). Final picture at the end of the treasure hunt (**d**)

The two modality-switching sessions were similar to previous modality-switching experiments with adults (see, e.g., Schaeffner et al., 2016a). However, an important difference was that, in line with other studies testing children of this age group with computer-based tasks (e.g., Atkinson et al., 2019), the modality-switching task was framed by a cover story, in order to strengthen children’s motivation. That is, at the beginning of the first modality-switching session, an introduction was given in which two protagonists, “Anna” and “Paul”, were introduced, and children were told that they had to help them on a treasure hunt (for selected pictures of the cover story, see Fig. 2). The second switching session again began with a welcome from the two protagonists, and it ended with the opening of a treasure box (see Fig. 2d). Between the different blocks of the sessions, the two protagonists provided motivating feedback such as “Well done!” or “We’re already halfway there!”, and a visual overview was shown of the number of blocks already completed, and those still to be completed (see Fig. 2b and c).

After the cover story in Session I or after the welcoming in Session II, each modality-switching session started with two single blocks (each including 16 trials), in which the two relevant modality mappings were used in isolation. This was necessary in order to calculate mixing costs, as well as to detect any general processing benefits for compatible compared to incompatible modality mappings. That is, the compatible switching session started with the auditory-vocal and the visual-manual single block (order counterbalanced across participants) before starting switching between compatible mappings. Similarly, the incompatible switching session started with the auditory-manual and visual-vocal single block (order counterbalanced across participants) before starting switching between incompatible modality mappings (see Fig. 1). After completing the two single blocks, children continued with three associated switching blocks (two blocks including 24 trials and one block including 16 trials^2^), in which they switched between the previously practiced modality mappings (e.g., between auditory-vocal and visual-manual in the compatible switching session; see Fig. 1). For switching, no instructional cues were needed because the required response (manual or vocal) was always determined by the input modality (auditory or visual). Additionally, there were eight demo trials before each single block, in which the experimenter demonstrated the task, followed by eight practice trials before each single block and before the first switching block of each session, in which the child practiced the task. If the child was still very unsure about the task or if there were more than 50% incorrect responses in the practice block, the practice trials were repeated. Altogether, there were 16 demo trials, at least 24 practice trials, and 96 experimental trials in each switching session.

Each trial started with the onset of the stimulus. Stimuli were presented in random order with the restriction that each stimulus was presented with the same frequency and that it was not directly repeated in two consecutive trials. The presentation of the stimuli lasted until a response was made or until 4000 ms had passed. During the presentation of the auditory stimuli, a blank screen was provided. The response-stimulus interval (RSI) was 2000 ms in the case of a correct response, so that a blank screen was presented for 2000 ms before the next trial started. In the event of an error, children received bimodal feedback consisting of a red dot in the center of the screen and a 202 Hz tone presented at the same time. Both were presented for 500 ms, which prolonged the RSI to 2500 ms in case of an error.

#### Design

For the analyses of single blocks, the independent within-subject variable was modality compatibility (compatible vs. incompatible). For the switching blocks, the independent within-subject variables were modality transition (repetitions in mixed-task blocks vs. single-task blocks for the mixing-costs analysis; switch vs. repetition in mixed-task blocks for the switch-costs analysis) and modality compatibility (compatible vs. incompatible). The dependent variables were RTs and error rates. All analyses were calculated at α = 0.05. Note that for data analyses, mean RTs and error rates were collapsed across the two compatible modality mappings (i.e., visual-manual vs. auditory-vocal) and across the two incompatible modality mappings (i.e., visual-vocal vs. auditory-manual). That is, the main effect of modality compatibility describes the difference in RTs and error rates between the average of the two compatible modality mappings and the average of the two incompatible mappings. Thus, our modality-compatibility contrast is independent of the influences of the individual modalities, but actually focuses on the mapping of input and output modalities, since both switching between compatible mappings and switching between incompatible mappings involves all four modalities.

### Results and discussion

The practice trials and the first trial of each block were discarded from all analyses. All responses given in the first 50 ms after stimulus onset were excluded because they were most likely voice-key artefacts (3.4%). For the identification of outliers, RTs of all trials were z-transformed for each subject separately, and trials with a z-score of −3/+3 were discarded as outliers (2.0%). Additionally, error trials and immediately subsequent trials were excluded from all analyses of RTs (14.4%). All mean RTs and error rates of each individual cell of the repeated-measures analysis of variance (ANOVAs) are presented in Table 1.

**Table 1:** Mean reaction times (RTs) and error rates (ERs) in experiment 1 during single-task and mixed-task performance, as a function of modality compatibility (i.e., compatible auditory–vocal and visual–manual mappings vs. incompatible auditory–manual and visual–vocal mappings), and transition type (modality switch vs. repetition)

		Mixed-task					
	Single-task	Repetition	Switch		Switch costs	Mixing costs	
**RTs**							
compatible	914	1159	1283		124	245	
incompatible	882	1260	1438		178	378	
**ERs**							
compatible	3.7	6.1	5.8		−0.3	2.4	
incompatible	3.4	4.7	7.7		3.0	1.3	

#### Single-task analysis

An ANOVA on single blocks with the independent within-subject variable modality compatibility (compatible vs. incompatible) revealed neither a significant effect of modality compatibility for RTs, *F*(1, 31) = 1.36; *p* =.252; η_p_² = 0.04, nor for error rates, *F*(1, 31) = 0.17; *p* =.687; η_p_² = 0.01. RTs were numerically even longer (914 ms versus 882 ms) and error rates even higher (3.7% versus 3.4%) for compatible compared to incompatible modality mappings (see Table 1), which rules out a general processing benefit for compatible modality mappings.

#### Mixing-costs analysis

**Fig. 3:**
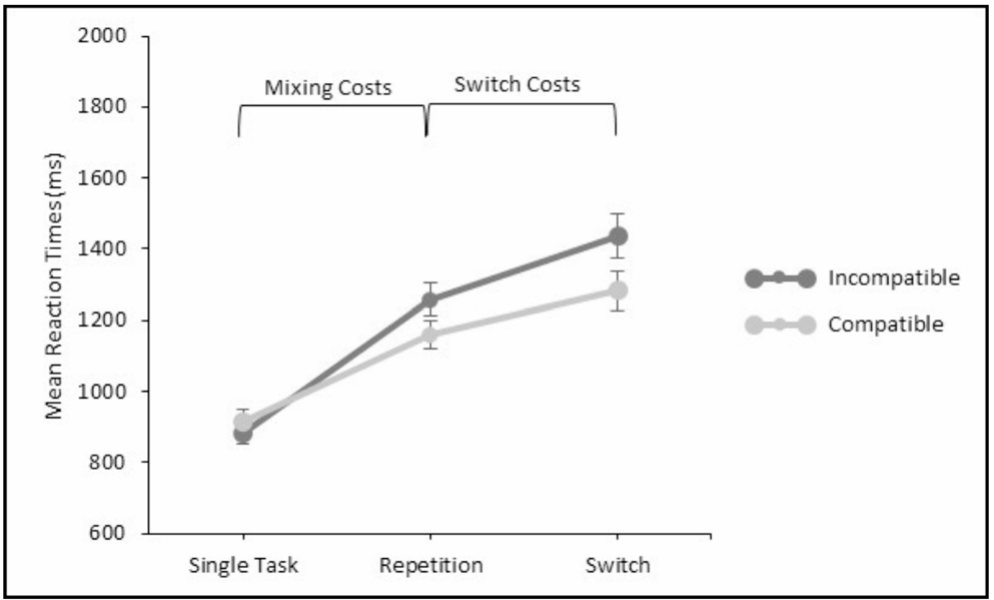
Mean reaction times in Experiment 1 for compatible modality mappings (i.e., auditory–vocal and visual–manual) and incompatible modality mappings (i.e., auditory–manual and visual–vocal). Error bars indicate the standard error of the mean

A 2 × 2 repeated measures ANOVA with the independent within-subject variables modality transition (repetitions in mixed-task blocks vs. single-task blocks) and modality compatibility (compatible vs. incompatible) yielded a significant main effect of modality transition in the RTs data, *F*(1, 31) = 158.89; *p* <.001; η_p_² = 0.84, revealing longer RTs in repetition trials in mixed-task blocks (1210 ms) compared to single-task blocks (898 ms; see Fig. 3). The main effect of modality compatibility was not significant, *F*(1, 31) = 1.74; *p* =.196; η_p_² = 0.05, but there was a significant interaction between modality transition and modality compatibility, *F*(1, 31) = 7.96; *p* =.008; η_p_² = 0.20, indicating significantly higher mixing costs for incompatible (378 ms) than for compatible modality mappings (245 ms).

In the error rates, there was also a significant main effect of modality transition, *F*(1, 31) = 7.20; *p* =.012; η_p_² = 0.19. Neither modality compatibility, *F*(1, 31) = 1.51; *p* =.229; η_p_² = 0.05, nor the interaction was significant, *F*(1, 31) = 0.95; *p* =.338; η_p_² = 0.03.^3^

#### Switch-costs analysis

A 2 × 2 repeated measures ANOVA with the independent within-subject variables modality transition (switches vs. repetitions) and modality compatibility (compatible vs. incompatible) yielded a significant main effect of modality transition in the RTs data, indicating significant modality switch costs, *F*(1, 31) = 30.08; *p* <.001; η_p_² = 0.49. That is, RTs were significantly longer in modality switch trials (1361 ms) than in repetition trials (1210 ms; see Fig. 3). The main effect of modality compatibility was also significant, *F*(1, 31) = 9.54; *p* =.004; η_p_² = 0.24. RTs were longer for switching between incompatible mappings (1349 ms) than for switching between compatible mappings (1221 ms; see Fig. 3). The interaction between modality transition and modality compatibility was not significant, *F*(1, 31) = 1.34; *p* =.256; η_p_² = 0.04, indicating no significant difference in switch costs for compatible and incompatible switching. Numerically, however, switch costs were higher for switching between incompatible modality mappings (178 ms) compared to compatible switching (124 ms).

In the error data, there was no significant effect of modality transition, *F*(1, 31) = 2.14; *p* =.153; η_p_² = 0.07. Numerically, error rates were higher for modality switching (6.8%) than in modality repetitions (5.4%). Modality compatibility was not significant, *F*(1, 31) = 0.04; *p* =.840; η_p_² = 0.00, but numerically, error rates were higher in incompatible modality mappings (6.2%) than in compatible mappings (6.0%). The interaction between modality transition and modality compatibility was significant, *F*(1, 31) = 4.31; *p* =.046; η_p_² = 0.12, indicating significantly higher switch costs for switching between incompatible than for switching between compatible modality mappings. Specifically, switch costs for incompatible switching were at an error rate of 3.0%, whereas there was even a slight switching advantage for compatible switches (−0.3% less error in switch than in repetition trials, see Table 1).^4^

#### Summary

Experiment 1 provides the first data on modality-compatibility effects on children’s modality switching. While the analysis of single-task performance did not reveal a significant effect of modality compatibility, so that a general processing benefit for compatible modality mappings can be ruled out, we found a significant effect of modality compatibility on mixing costs. Mixing costs were higher for incompatible mappings than for compatible mappings (in the RTs data as well as in a combined consideration of RTs and error rates in the form of the IES). On switch costs, in contrast, the effect of modality compatibility was very small. Even though there was a significant interaction of transition and modality compatibility in the switch-costs analysis of the error rates, the interaction was neither significant in the analysis of RTs data nor in the analysis of the IES. This represents an important new finding, as previous modality-switching studies with adults (e.g., Schaeffner et al., 2016a, b, 2018a; Stephan & Koch, 2010, 2011) have typically shown very stable effects of modality compatibility on switch costs (see Schacherer & Hazeltine, 2019, for an exception in the absence of conceptual overlap across tasks). Thus, one might argue that children’s modality switching is less influenced by modality compatibility than modality switching of adults, because children have less experience with response effects than adults, who have many years of response-effect learning, and who thus experience greater crosstalk than children when they switch between incompatible modality mappings. However, it should be noted that the children in the present study were between six and ten years old, so that response effects should largely be internalized, making the presence of modality-compatibility effects plausible. Therefore, we assume that the very small modality compatibility effects are not necessarily age-specific, but may also be influenced by the type of input. If, as in adults, the strength of these effects varies depending on input type, they may be more pronounced during the processing of verbal input. (see Schaeffner et al., 2018b). Verbal input—and even verbal output—that plays an essential role in children’s language development from a very early age includes the integrated processing of spoken language and gestures (see, e.g., Capirci et al., 1996; Lüke et al., 2017). Therefore, we assume that modality-compatibility effects in children might be especially pronounced when switching between spoken language and gestures. To gain more clarity on this issue, we conducted Experiment 2.

## Experiment 2

In Experiment 2, we aimed to repeat Experiment 1, replacing the nonverbal presentation of the concepts with spoken words and gestures. We expected modality-compatibility effects in terms of longer RTs, higher error rates, higher mixing costs, as well as higher modality switch costs for incompatible modality mappings than for compatible ones.

### Method

#### Participants

Thirty-two new participants (who did not participate in Experiment 1) were tested in Experiment 2 (*M*_age_ = 8.4 years; *SD* = 1.3 years; range = 6.3–10.4; gender = 9 female). All children were German speakers with a typical language development, which was ensured by conducting the same standardized language tests in the Pretest as reported for Experiment 1. No child had experience with sign language. Thirty-one of them were right-handed. Parents reported no neurological, psychiatric, or developmental disorders^5^, as well as normal hearing acuity and normal or corrected-to-normal visual acuity. Parental consent was received for all participants. Recruiting and compensation were identical to Experiment 1.

#### Task and procedure

In Experiment 2, children went through the three sessions in the same manner as in Experiment 1. The time interval between sessions ranged from 1 to 35 days (*M* = 9 days). The main difference compared to Experiment 1 concerned the presentation of the concepts in Sessions I and II. Specifically, the less verbal presentation of Experiment 1 was replaced by a more verbal presentation. That is, the same concepts as in Experiment 1 were now presented either as spoken words (i.e., auditory input) or as gestures (i.e., visual input). The words were spoken by a female voice with neutral accent. They were recorded in advance and the sound files were edited with the software Audacity (http://audacity.sourceforge.net/). During the experiment, the words were presented over the same loudspeakers as in Experiment 1 at an average volume of 55 dB, and with a controlled duration (between 400 and 900 ms). The gestures were presented as silent video clips. Each video clip displayed the upper body of a woman performing a gesture with both hands and arms in front of a white background, and with a controlled duration (between 2110 and 3510 ms). Gestures were all based on German Sign Language. To ensure that the gestures could be clearly assigned to the desired concepts, 20 independent adult raters assessed them all in advance. Table 2 gives an overview of all concepts and corresponding gestures used in Experiment 2.

**Table 2:**
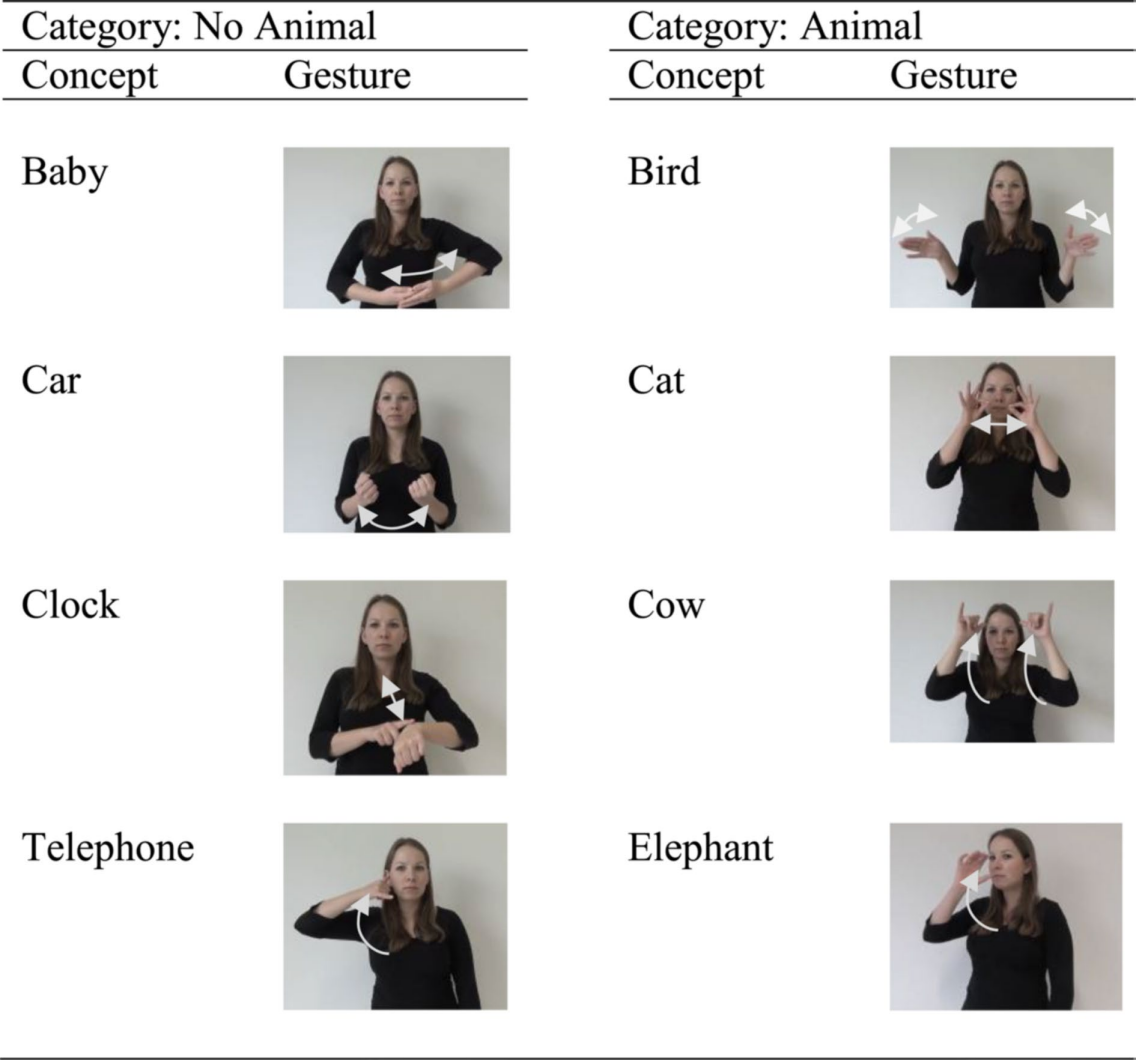
Excerpts of the gesture videos of experiment 2

#### Design

Similar to Experiment 1, the independent within-subject variable for the analyses of single blocks was modality compatibility (compatible vs. incompatible). For the switching blocks, the independent within-subject variables were modality transition (repetitions in mixed-task blocks vs. single-task blocks for the mixing-costs analysis; switch vs. repetition in mixed-task blocks for the switch-cost analysis), and modality compatibility (compatible vs. incompatible). The dependent variables were RTs and error rates. All analyses were calculated at α = 0.05.

### Results and discussion

Data analyses followed the same procedure as in Experiment 1. Practice trials, the first trial of each block, as well as all responses given in the first 50 ms after stimulus onset (4.1%), and trials with a z-score of −3/+3 (1.3%) were discarded from all analyses. Additionally, error trials and immediately subsequent trials were excluded from all analyses of RTs (14.7%). All mean RTs and error rates of each individual cell of the repeated measures ANOVAs are presented in Table 3.

**Table 3:** Mean reaction times (RTs) and error rates (ERs) in Experiment 2 during single-task and mixed-task performance, as a function of modality compatibility (i.e., compatible auditory–vocal and visual–manual mappings vs. incompatible auditory–manual and visual–vocal mappings), and transition type (modality switch vs. repetition)

		Mixed-task			
	Single-task	Repetition	Switch	Switch costs	Mixing costs
**RTs**					
compatible	1337	1455	1552	97	118
incompatible	1249	1543	1711	168	294
compatible	3.7	2.7	4.3	1.6	-1
incompatible	5.2	5.2	9.0	3.8	0

#### Single-task performance

An ANOVA on single-task performance with the independent within-subject variable modality compatibility (compatible vs. incompatible) revealed a significant effect of modality compatibility for RTs, *F*(1, 31) = 6.73; *p* =.014; η_p_² = 0.18, indicating longer RTs for compatible modality mappings (1337 ms), compared to incompatible mappings (1249 ms). The difference in error rates was not significant, *F*(1, 31) = 2.36; *p* =.134; η_p_² = 0.07, and error rates were numerically even higher for incompatible (5.2%) compared to compatible modality mappings (3.7%; see Table 3), indicating a speed-accuracy tradeoff.^6^

#### Mixing-costs analysis

**Fig. 4:**
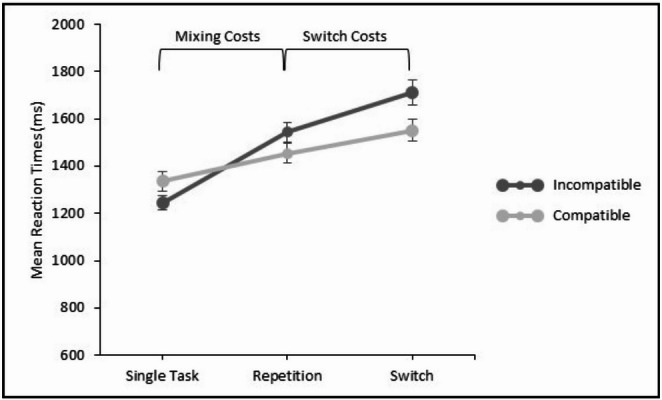
Mean reaction times in Experiment 2 for compatible modality mappings (i.e., auditory–vocal and visual–manual) and incompatible modality mappings (i.e., auditory–manual and visual–vocal). Error bars indicate the standard error of the mean

A 2 × 2 repeated measures ANOVA with the independent within-subject variables modality transition (repetitions in mixed-task blocks vs. single-task blocks) and modality compatibility (compatible vs. incompatible) yielded a significant main effect of modality transition in the RTs data, *F*(1, 31) = 113.83; *p* <.001; η_p_² = 0.79, revealing longer RTs in repetition trials in mixed-task blocks (1499 ms), compared to single-task blocks (1293 ms; see Fig. 4). There was no main effect of modality compatibility, *F*(1, 31) = 0.00; *p* =.955; η_p_² = 0.00, but there was a significant interaction between modality transition and modality compatibility, *F*(1, 31) = 23.52; *p* <.001; η_p_² = 0.43. That is, mixing costs were significantly higher for incompatible (294 ms) than for compatible modality mappings (118 ms).

In the error rates, there was no significant main effect of modality transition, *F*(1, 31) = 0.40; *p* =.530; η_p_² = 0.01, but a significant main effect of modality compatibility, *F*(1, 31) = 11.31; *p* =.002; η_p_² = 0.27, indicating higher error rates in incompatible (5.2%), than in compatible modality mappings (3.2%). The interaction was not significant, *F*(1, 31) = 0.44; *p* =.511; η_p_² = 0.01.^7^

#### Switch-costs analysis

A 2 × 2 repeated measures ANOVA with the independent within-subject variables modality transition (switches vs. repetitions) and modality compatibility (compatible vs. incompatible) yielded a significant main effect of modality transition in the RTs data, indicating modality switch costs, *F*(1, 31) = 40.33; *p* <.001; η_p_² = 0.57. That is, RTs were significantly longer in modality switch trials (1632 ms) than in repetition trials (1499 ms; see Fig. 4). The main effect of modality compatibility was also significant, *F*(1, 31) = 16.66; *p* <.001; η_p_² = 0.35. RTs were longer in incompatible trials (1627 ms) than in compatible trials (1504 ms). Moreover, the interaction between modality transition and modality compatibility was significant, *F*(1, 31) = 6.42; *p* =.017; η_p_² = 0.17, indicating higher switch costs for switching between incompatible modality mappings (168 ms) compared to switching between compatible mappings (97 ms; see Fig. 4).

The analysis of the error rates revealed a significant main effect of modality transition, *F*(1, 31) = 8.67; *p* =.006; η_p_² = 0.22, indicating higher error rates in modality switches (6.7%) compared to modality repetitions (3.9%). The main effect of modality compatibility was also significant, *F*(1, 31) = 16.24; *p* <.001; η_p_² = 0.34, showing higher error rates in incompatible modality mappings (7.1%) than in compatible mappings (3.5%). The interaction between modality transition and modality compatibility was not significant, *F*(1, 31) = 2.28; *p* =.141; η_p_² = 0.07.^8^

#### Summary

In contrast to Experiment 1, Experiment 2 revealed strong effects of modality compatibility on both mixing costs and switch costs. Mixing costs as well as switch costs were significantly higher for incompatible modality mappings than for compatible ones in the RTs data. Regarding error rates, both performance costs were not significantly influenced by modality compatibility. However, post-hoc analyses based on IES demonstrated significant effects for both mixing and switch costs. Thus, the influence of modality compatibility on modality switching seems to be more pronounced in Experiment 2 than in Experiment 1. To gain further insights into the differences between the two experiments, we conducted a supplementary analysis, in which we directly compared Experiment 1 and 2 by including “experiment” as a between-subjects variable in our analysis.

## Supplemental analysis

We ran a 2 × 2 mixed ANOVA with the independent within-subject variable modality compatibility (compatible vs. incompatible) and the between-subject variable experiment (Experiment 1 vs. Experiment 2). The dependent variables were proportional mixing and proportional switch costs, because the actual RTs data and error rates were not comparable across the two experiments due to the differing input (i.e., pictures and tones in Experiment 1 versus gestures and spoken words in Experiment 2). The arguably more conclusive values are the mixing and switch cost. To make sure that the effects of sensory-motor modality compatibility on switch costs are not just due to proportionally higher costs in modality mappings associated with longer RTs, we calculated proportional mixing and proportional switch costs and used them as the dependent variable of the supplemental analysis. All calculations were based on the IES in order to integrate both RTs and error rates in the analyses. Specifically, proportional mixing costs were calculated by dividing the mean IES of mixing costs by the mean IES of single-task performance. Proportional switch costs were calculated by dividing the mean IES of switch costs by the mean IES of repetition trials.

### Mixing-costs analysis

The analysis of proportional mixing costs yielded a significant main effect of modality compatibility, *F*(1, 62) = 17.31; *p* <.001; η_p_² = 0.22, revealing higher proportional mixing costs for incompatible modality mappings (36.4%) compared to compatible ones (20.2%). The main effect of experiment was also significant, *F*(1, 62) = 32.05; *p* <.001; η_p_² = 0.34, indicating higher proportional mixing costs in Experiment 1 (40.2%) than in Experiment 2 (16.44%). The interaction was not significant, *F*(1, 62) = 0.01; *p* =.928; η_p_² = 0.00.

### Switch-costs analysis

The analysis of proportional switch costs yielded a significant main effect of modality compatibility, *F*(1, 62) = 9.35; *p* =.003; η_p_² = 0.13, revealing higher proportional switch costs for switching between incompatible modality mappings (17.9%) compared to switching between compatible mappings (9.6%). There was neither a significant main effect of experiment, *F*(1, 62) = 0.52; *p* =.472; η_p_² = 0.01, nor a significant interaction, *F*(1, 62) = 0.15; *p* =.702; η_p_² = 0.00.

### Summary

The supplemental analyses demonstrate that the only significant difference between Experiment 1 and Experiment 2 was in the magnitude of mixing costs (i.e., higher mixing costs in Experiment 1 than in Experiment 2). Regarding the influence of modality compatibility, however, no significant difference between the two experiments was found.

## General discussion

The present study aimed to examine the role of modality compatibility in children’s modality switching. In Experiment 1, we found significantly higher mixing costs for incompatible mappings compared to compatible ones. Switch costs were only marginally affected by modality compatibility in Experiment (1) In Experiment 2, both mixing costs and switch costs were significantly higher for incompatible than for compatible mappings. A supplemental analysis directly comparing the two experiments revealed that the difference in switch costs between the experiments (i.e., a significant effect of modality compatibility on switch costs in Experiment 2 but only a marginal influence in Experiment 1) was not statistically significant. The only significant difference between the two experiments was in the magnitude of mixing costs. Specifically, mixing costs were significantly higher in Experiment 1 than in Experiment (2) In the following, we will discuss the implications of these findings for understanding modality-specific effects on children’s cognitive control mechanisms and what they suggest about the role of modality compatibility in children’s language processing.

### Modality-specific effects on children’s cognitive control mechanisms

Cognitive control processes are often studied by task-switching studies (for a review, see Koch et al., 2018), which often include an analysis of mixing costs as well as an analysis of switch costs. Both types of performance costs allow conclusions to be drawn about different cognitive mechanisms. While mixing costs are defined as more global performance costs that arise when multiple tasks are relevant, switch costs are defined as more local performance costs due to switching from one task to another. Thus, mixing costs are seen as a marker for a higher working memory load resulting from maintaining two task sets in parallel (e.g., Los, 1996). In contrast, switch costs are seen as a marker for cognitive control mechanisms necessary to manage competition between task sets when the current task must be reconfigured during a switch from one task to another (Monsell, 2003). While previous studies show that both mixing costs and switching costs already occur in childhood (e.g., Reimers & Maylor, 2005; Vernucci et al., 2023), the present study further shows that both children’s mixing costs (see Experiments 1 and 2) and switch costs (see Experiment 2) can be influenced by modality compatibility. However, the present study also revealed differences between these two types of performance costs, which will be discussed below.

On the one hand, both experiments revealed significant mixing costs. This means that in both experiments, children experienced a higher working memory load in switching blocks than in single blocks. However, the direct comparison of the two experiments in the supplemental analysis demonstrated that mixing costs were significantly higher in Experiment 1 than in Experiment 2. Since the only difference between the two experiments was the type of input, we can conclude that it requires higher working memory load to keep nonverbal input in the form of pictures and sounds activated in parallel than verbal input in the form of gestures and spoken words. This represents an interesting finding, which is in line with the assumption of a specific relationship between sign language and spoken language. In contrast to two spoken languages, sign language and spoken language can be produced in parallel in terms of so-called code-blending (Emmorey et al., 2008). As both languages are clearly assigned to different modalities, and as there is no phonological overlap, both languages can be activated in parallel with little competition (see also Schaeffner et al., 2017). While code-blending is very natural in spontaneous language production of bimodal bilinguals (i.e., mostly hearing individuals who are proficient in a spoken and a sign language), the integration of gestural information into spoken language is also relevant for children growing up in a spoken-language environment. Even if they are not familiar with sign language, they are often confronted with spoken language enriched with gestural information, which clearly helps rather than hinders language processing (for an overview, see Rowe et al., 2022). This experience could make it particularly easy for children to keep spoken language and gestural information activated in parallel in working memory, resulting in lower mixing costs in Experiment 2 compared to Experiment 1.

On the other hand, switch costs did not significantly differ between Experiment 1 and Experiment 2 (see supplemental analysis). That is, switching between nonverbal pictures and sounds did not evoke higher switch costs than switching between gestures and spoken words. Thus, task-set reconfiguration while switching between the different modality mappings required a similar amount of cognitive control in both experiments. Separate analyses of Experiment 1 and 2, however, revealed a significant influence of modality compatibility on switch costs in Experiment 2 but only a marginal influence in Experiment 1. That is, switch costs for switching between gestures and spoken words (Experiment 2) were significantly influenced by modality compatibility, similar to modality switching of adults (e.g., Stephan & Koch, 2010, 2011; Schaeffner et al., 2016a). In contrast, switch costs for switching between nonverbal pictures and sounds (Experiment 1) were only marginally influenced by modality compatibility.

One might argue that the marginal influence of modality compatibility in Experiment 1 reflects age-related differences in cognitive control mechanisms, given that previous studies with adults typically report robust modality-compatibility effects (e.g., Stephan & Koch, 2010, 2011; Schaeffner et al., 2016a). However, this interpretation should be treated with caution, as the difference compared to adults emerges only in switch costs, not in mixing costs—an observation that appears somewhat at odds with earlier research on age-related effects (e.g., Peng et al., 2018b, found no age-related differences in either cost type; Reimers & Maylor, 2005, reported age effects in mixing costs but not in switch costs). Moreover, a direct comparison between the present study and previous adult studies is limited by methodological differences, such as varying numbers of trials and different types of stimulus material. Finally, it should be noted that there was no difference in the overall magnitude of switch costs —that is, significant modality switch costs were also found in Experiment 1, consistent with findings in adult populations. The differences lay only in the influence of modality compatibility on those costs. We therefore assume that the marginal effect of modality compatibility on switch costs is more likely due to input type–specific variation than to age-related differences in cognitive control mechanisms.

In line with previous experiments with adults, the present study demonstrates that modality compatibility effects on switch costs can be increased with verbal input compared to nonverbal input (Schaeffner et al., 2018b). One possible explanation for stronger effects of modality compatibility for verbal input might be the activation of the mirror neuron system. The mirror neuron system represents a set of neural regions that underlie imitative processes (e.g., Iacoboni, 2009), always activated when an individual performs intentional actions or when they observe another person’s intentional action (e.g., Decety & Grèzes, 1999). As verbal input in terms of gestures and spoken words represents intended actions that are learned by observation and imitation, the mirror neuron system should be activated when participants process verbal input, resulting in an activation of compatible modality mappings. For example, listening to spoken words leads to covert imitation of the articulatory movements of the speaker (for a review see Galantucci et al., 2006), activating the compatible auditory-vocal modality mapping. In contrast, listening to nonverbal sounds should be less associated with any intended action and less associated with any vocal output, resulting in less strong activations of the compatible auditory-vocal mappings. Thus, crosstalk between the two incompatible modality mappings should be stronger for the processing of verbal input than for processing nonverbal input, resulting in stronger modality-compatibility effects on switch costs for verbal input. However, it should be noted that the apparent differences in modality-compatibility effects on switch costs between verbal input (Experiment 2) and nonverbal input (Experiment 1) were not confirmed by the supplementary analysis. Further research is therefore needed to achieve a more comprehensive understanding of the factors that modulate the influence of modality compatibility on children’s modality switching.

### Modality compatibility in children’s language processing

Beyond suggesting potential differences between verbal and nonverbal input processing, the results of the present study extend previous findings on modality switching (e.g., Schaeffner et al., 2016a, 2018b) by clearly demonstrating that modality compatibility plays a role in language processing—not only in adults, but also in children. Assuming that modality-compatibility effects arise from increased crosstalk when switching between incompatible modality mappings, due to lifelong response–effect learning, the current findings demonstrate that children aged six to ten years have already internalized these response effects in a way comparable to adults.

Taking this into account, a number of new questions arise regarding modality-specific effects in children’s language processing—particularly in educational and therapeutic contexts. For example, the present findings may contribute to the discussion on modality-specific aspects of dyslexia (e.g., Ramus, 2003). Sensory processing deficits—such as general auditory deficits (e.g., McWeeny & Norton, 2024), visual processing impairments (e.g., Kristjánsson & Sigurdardottir, 2023), or even motor deficits (e.g., Decarli et al., 2024)—are currently being debated as possible explanations for dyslexia. However, potential interactions between sensory and motor processing in the context of modality compatibility have largely been overlooked. Although previous research has shown that children with dyslexia struggle with shifting attention between modalities (e.g., Harrar et al., 2014), no study to date has investigated how they manage switching between compatible and incompatible modality mappings.

Another important question arising from the present findings concerns the role of modality compatibility in the context of Developmental Language Disorder (DLD). Numerous studies have examined task-switching performance in children with DLD (e.g., Boerma et al., 2022; Giandomenico et al., 2023) to gain insights into the cognitive mechanisms underlying the disorder. However, to date, no study has specifically investigated modality switching in this population. This gap is particularly noteworthy, given that children with DLD are known to rely more heavily on gestures and to produce more gestures than typically developing peers in order to compensate for deficits in spoken language (e.g., Iverson & Braddock, 2011; Lavelli & Majorano, 2016; Mainela-Arnold et al., 2014). This compensation strategy likely results in frequent switching between modalities even from a very early age. It thus raises the question of whether children with DLD might actually be more proficient in modality switching than typically developing children—and whether their switching behaviour is similarly affected by modality compatibility.

### Conclusion

In summary, the present study is the first to demonstrate that children’s modality switching is influenced by modality compatibility. Across two modality-switching experiments, modality compatibility effects were observed in performance costs, albeit with some variation between the experiments. While significant effects of modality compatibility on mixing costs were found in both experiments, switch costs were only significantly influenced in Experiment 2, when children processed verbal input. In contrast, switching between nonverbal stimuli—pictures and sounds—in Experiment 1 resulted in only marginal modality compatibility effects on switch costs. These findings introduce a novel perspective on children’s language processing and may serve as a foundation for further basic research, as well as for applied investigations in therapeutic and educational contexts.

## Data Availability

The original data can be found at [https://osf.io/mwsxj/?view\_only=33491348ec9a49e8b528515efa02a779](https:/osf.io/mwsxj/?view_only=33491348ec9a49e8b528515efa02a779).

## Footnotes

1 Effects of modality compatibility on modality switching in studies with adults have been repeatedly replicated with sample sizes of 16 participants (e.g., Schaeffner et al., 2016a; Stephan & Koch, 2010, 2011). For the present study, however, some methodological adjustments were necessary, such as a reduction in the number of trials, in order to make the task suitable for children. Using GPower 3.1 (Faul et al., 2009) we calculated that a sample size of 32 participants allowed us to detect a medium effect (Cohen’s f = 0.25) at a significance level of α = 0.05 with a power of 0.92. The power analysis was based on a 2 × 2 repeated measures ANOVA with two within-subject variables, which was relevant for the planned mixing-costs and switch-costs analyses.

2 Altogether, there were 16 different stimuli in the experiment (i.e., eight different concepts presented either auditorily or visually). All stimuli were presented the same number of times within each condition. Pilot tests showed that some children had difficulty concentrating during switching when a block contained 32 trials (i.e., when each stimulus was presented twice). Therefore, we decided to shorten the blocks: two longer blocks with 24 trials each, and one short block with16 trials. The short block contained all 16 stimuli. The longer blocks included all 16 stimuli, plus an additional half of the stimulus set (i.e., eight stimuli), resulting in 24 trials per block. The second long block always contained the eight stimuli not repeated in the first long block, so that, in the end, each stimulus was presented four times within the entire condition.

3 Since the interaction between modality transition and modality compatibility was significant in the RTs data but not in the error rates, we performed a post-hoc analysis based on inverse efficiency scores (IES), which represent an integrated speed-accuracy measure (e.g., Liesefeld & Janczyk, 2019) by dividing RTs in each condition by accuracy (1 – percentage of error rates). In this analysis, there was a significant effect of modality transition, *F*(1, 31) = 126.78; *p* <.001; η_p_² = 0.80, revealing higher IES in repetition trials in mixed-task blocks (1284 ms) compared to single-task blocks (936 ms). The main effect of modality compatibility was not significant, *F*(1, 31) = 0.48; *p* =.495; η_p_² = 0.02, but there was a significant interaction between modality transition and modality compatibility, *F*(1, 31) = 5.05; *p* =.032; η_p_² = 0.14, indicating significantly higher mixing costs for incompatible mappings (412 ms) than compatible mappings (285 ms).

4 Since the interaction between modality transition and modality compatibility was not significant in the RTs data but in the error data, we performed a post-hoc analysis based on IES to get a clearer picture of the influence of modality compatibility on modality switch costs. The analysis revealed a significant main effect of modality transition, *F*(1, 31) = 24.71; *p* <.001; η_p_² = 0.44, indicating significantly higher IES in modality switch trials (1475 ms) than in repetition trials (1284 ms). The main effect of modality compatibility was also significant, *F*(1, 31) = 7.56; *p* =.010; η_p_² = 0.20. The IES were higher in incompatible trials (1452 ms) than in compatible trials (1307 ms). The interaction between modality transition and modality compatibility was not significant, *F*(1, 31) = 3.43; *p* =.073; η_p_² = 0.10.

5 One child had a diagnosis of ADHD and one child had a diagnosis of Dyslexia. As the two diagnoses only became known after the study was completed, we decided not to exclude the children retrospectively. However, to ensure that the two diagnoses had no influence on relevant effects, we recalculated the analyses excluding the two children. No change was found in the data pattern.

6 Post-hoc analyses using the IES revealed no significant effect of modality compatibility, *F*(1, 31) = 3.23; *p* =.082; η_p_² = 0.09, further confirming a speed-accuracy tradeoff in single-task performance of Experiment 2.

7 Analogous to the analyses of Experiment 1, we conducted a post-hoc analysis based on IES, which revealed a significant effect of modality transition, *F*(1, 31) = 78.20; *p* <.001; η_p_² = 0.72, revealing higher IES in repetition trials in mixed-task blocks (1568 ms) compared to single-task blocks (1355 ms). The main effect of modality compatibility was not significant, *F*(1, 31) = 0.77; *p* =.387; η_p_² = 0.02, but there was a significant interaction between modality transition and modality compatibility, *F*(1, 31) = 19.47; *p* <.001; η_p_² = 0.39, indicating significantly higher mixing costs for incompatible (316 ms) than for compatible modality mappings (110 ms).

8 To gain more clarity about the interaction, which was significant in the RTs data but not in the error rates, we performed a post-hoc analysis based on IES. The analysis revealed a significant main effect of modality transition, *F*(1, 31) = 44.02; *p* <.001; η_p_² = 0.59, indicating significantly higher IES in modality switch trials (1764 ms) than in repetition trials (1568 ms). The main effect of modality compatibility was also significant, *F*(1, 31) = 19.91; *p* <.001; η_p_² = 0.39. IES were longer in incompatible trials (1767 ms) than in compatible trials (1565 ms). The interaction between modality transition and modality compatibility was significant as well, F(1, 31) = 10.50; *p* =.003; η_p_² = 0.25, showing higher switch costs for switching between incompatible (263 ms) than for switching between compatible modality mappings (128 ms).
